# PlantAPA: A Portal for Visualization and Analysis of Alternative Polyadenylation in Plants

**DOI:** 10.3389/fpls.2016.00889

**Published:** 2016-06-21

**Authors:** Xiaohui Wu, Yumin Zhang, Qingshun Q. Li

**Affiliations:** ^1^Department of Automation, Xiamen UniversityXiamen, China; ^2^Key Laboratory of the Ministry of Education for Coastal and Wetland Ecosystems, College of the Environment and Ecology, Xiamen UniversityXiamen, China; ^3^Graduate College of Biomedical Sciences, Western University of Health SciencesPomona, CA, USA

**Keywords:** plant, alternative polyadenylation, database, web server, 3′ UTR, mRNA processing

## Abstract

Alternative polyadenylation (APA) is an important layer of gene regulation that produces mRNAs that have different 3′ ends and/or encode diverse protein isoforms. Up to 70% of annotated genes in plants undergo APA. Increasing numbers of poly(A) sites collected in various plant species demand new methods and tools to access and mine these data. We have created an open-access web service called PlantAPA (http://bmi.xmu.edu.cn/plantapa) to visualize and analyze genome-wide poly(A) sites in plants. PlantAPA provides various interactive and dynamic graphics and seamlessly integrates a genome browser that can profile heterogeneous cleavage sites and quantify expression patterns of poly(A) sites across different conditions. Particularly, through PlantAPA, users can analyze poly(A) sites in extended 3′ UTR regions, intergenic regions, and ambiguous regions owing to alternative transcription or RNA processing. In addition, it also provides tools for analyzing poly(A) site selections, 3′ UTR lengthening or shortening, non-canonical APA site switching, and differential gene expression between conditions, making it more powerful for the study of APA-mediated gene expression regulation. More importantly, PlantAPA offers a bioinformatics pipeline that allows users to upload their own short reads or ESTs for poly(A) site extraction, enabling users to further explore poly(A) site selection using stored PlantAPA poly(A) sites together with their own poly(A) site datasets. To date, PlantAPA hosts the largest database of APA sites in plants, including *Oryza sativa, Arabidopsis thaliana, Medicago truncatula*, and *Chlamydomonas reinhardtii*. As a user-friendly web service, PlantAPA will be a valuable addition to the community of biologists studying APA mechanisms and gene expression regulation in plants.

## Introduction

Messenger RNA (mRNA) 3′ end formation, including cleavage and polyadenylation, is a vital step in post-transcriptional processing in eukaryotic cells. Polyadenylation plays important roles in many mRNA functions, such as mRNA stability, exportation, and translation initiation (Xing and Li, 2011). Accumulating evidence suggests that polyadenylation has been widely employed by many plant and mammalian species, through alternative polyadenylation (APA), to produce mRNAs having different 3′ ends or encoding variable protein isoforms. Recent genomic studies have uncovered widespread occurrences of APA in different species. In animals, 70–79% of mammalian genes (Derti et al., 2012; Hoque et al., 2013) and about half of the genes in flies (Smibert et al., 2012) and zebrafish (Li et al., 2012; Ulitsky et al., 2012) have at least two poly(A) sites. In the green alga *Chlamydomonas reinhardtii* (Chlamy for short herein), APA affects transcripts from 33 to 68% of expressed genes (Shen et al., 2008b; Zhao et al., 2014; Bell et al., 2016). In higher plants, 64% of *Medicago truncatula* (referred as Medicago herein) genes possess more than one poly(A) site (Wu et al., 2014), and ~70% of annotated genes undergo APA in Arabidopsis and rice (Shen et al., 2008a, 2011; Wu et al., 2011; Sherstnev et al., 2012). As data accumulate, such increasingly high numbers of APA sites should be made accessible in a user-friendly manner to facilitate investigation of important biological questions.

Currently, several web services are available to access APA-related data, and each of these platforms has its own strengths and limitations. Databases such as PACdb (Brockman et al., 2005), polyA_DB (Zhang et al., 2005), and polyA_DB2 (Beaudoing and Gautheret, 2001; Lee et al., 2007) are available to query poly(A) sites for multiple organisms, especially in animals which were identified primarily through cDNAs and expressed sequence tags (ESTs). However, because of the limited collection of cDNAs and ESTs, these databases only catalog poly(A) sites or sequences in 3′ UTRs (Untranslated Regions) and therefore lack poly(A) sites located in introns or coding sequences (CDS) that have been increasingly discovered in recent transcriptome studies by second generation sequencing platforms. UTRdb (Grillo et al., 2010) describes curated sequences and functional motifs in 5′ and 3′ UTRs, but it provides no poly(A) site or poly(A) signal information. Two of the latest databases, APADB (Müller et al., 2014) and APASdb (You et al., 2014), provide poly(A) sites for coding and non-coding transcripts of animals, including human, mouse, chicken, and zebrafish, utilizing the 3′ end sequencing to identify poly(A) sites. However, although these databases contain an abundance of poly(A) sites at the whole genome level, datasets derived from animals are still limited. PolyADB (https://www.compbio.dundee.ac.uk/polyADB/) stores read alignments and poly(A) sites for human, chicken, and Arabidopsis using sequences from RNA-seq and direct sequencing, but it lacks datasets for other plant species and provides limited capability for data visualization and analysis. Recent studies have discovered an increasing number of APA genes in various plant species, including rice, Arabidopsis, Medicago, and Chlamy (Shen et al., 2008a; Wu et al., 2011, 2014; Sherstnev et al., 2012; Thomas et al., 2012; Zhao et al., 2014; Bell et al., 2016). However, the existing APA-related web services typically provide little or no integration with either poly(A) sites in plants or poly(A) site extraction, and no utilities for visualization or analysis are provided.

Here, we present an open-access web service called PlantAPA (http://bmi.xmu.edu.cn/plantapa) to query, visualize, and analyze genome-wide poly(A) sites derived from various data sources, including RNA-seq, ESTs, 454, and poly(A) tag (PAT) sequencing (Liu et al., 2015) for different plant species. PlantAPA provides various interactive and dynamic graphics and seamlessly integrates a popular genome browser, Jbrowse (Skinner et al., 2009), for profiling heterogeneous cleavage sites and quantifying expression patterns of poly(A) sites across different conditions. Moreover, PlantAPA details poly(A) sites in different genic regions (introns, CDS, 3′ UTRs, and 5′ UTRs), extended 3′ UTRs, intergenic regions, and ambiguous regions owing to alternative transcription or RNA processing. In particular, PlantAPA is capable of analyzing poly(A) site selection, 3′ UTR lengthening or shortening, and differential gene expression between conditions, making it more powerful for the study of APA mechanisms and APA-mediated gene regulation than the previous web services. Additionally, PlantAPA allows users to upload their own short reads or ESTs to extract poly(A) sites through the poly(A) site extraction pipeline. Therefore, users can retrieve, query, and analyze their own poly(A) sites with stored PlantAPA poly(A) sites to further explore poly(A) site selection across existing conditions and conditions provided by the user. Currently, PlantAPA provides poly(A) site datasets from four plant species, including rice, Arabidopsis, Medicago, and Chlamy, which were sequenced from different type of cells, tissues, and conditions. In the future, we will incorporate more plant species and poly(A) site datasets as more data become available. As a user-friendly web service, PlantAPA will be a valuable tool for the study of APA mechanisms in plants, not only by making high-quality poly(A) sites more accessible, but also by integrating many valuable add-ons to enhance the usability and functionality of our web service.

## Materials and methods

### Processing of sequences and genome annotations

**Figure 1:**
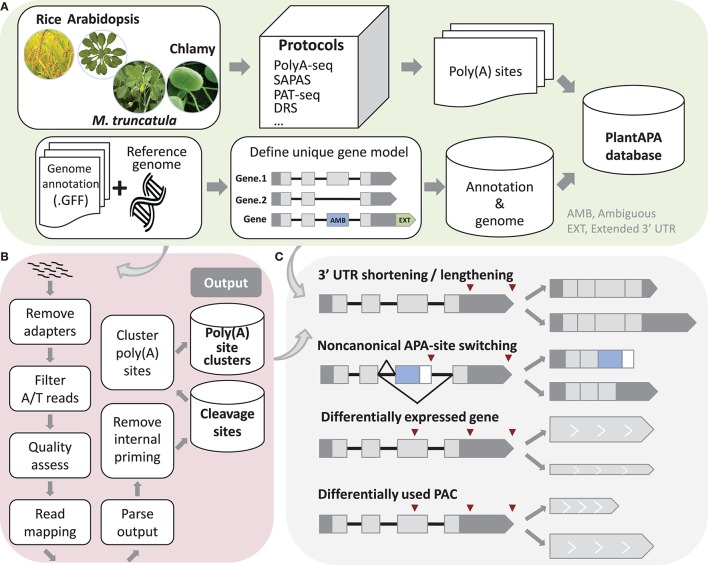
**Schema of methods used in PlantAPA. (A)** Processing of sequences and genome annotations. To determine unique gene models, ambiguous regions of genes with more than one annotated transcript are defined. Genes without annotated 3′ UTR are assigned a 3′ UTR with average 3′ UTR length, and annotated 3′ UTRs are extended by a fixed length. **(B)** Pipeline to detect poly(A) sites from next generation sequencing reads. **(C)** Schema of 3′ UTR lengthening and shortening, non-canonical APA site switching genes, differentially expressed genes, and differentially used PACs.

ESTs, raw reads from PAT-seq sequencing, and poly(A) sites from previous studies (Shen et al., 2008a; Wu et al., 2011, 2014; Thomas et al., 2012; Liu et al., 2014; Zhao et al., 2014; Bell et al., 2016) were processed and mapped to the latest reference genomes, and the coordinates of poly(A) sites were recorded (Figure 1A). Poly(A) sites of Arabidopsis were from leaf, seed, and root tissues of wild type (WT) and mutant *oxt6* (Wu et al., 2011; Thomas et al., 2012; Liu et al., 2014). Poly(A) sites sequenced from PAT-seq sequencing of Medicago were from leaf tissues (Wu et al., 2014). Rice poly(A) sites from ESTs (Shen et al., 2008a) were re-annotated with the MSU v7 genome annotation (Kawahara et al., 2013). Poly(A) sites of Chlamy collected from PAT-seq sequencing (Bell et al., 2016), ESTs, 454, and Illumina sequencing data (Zhao et al., 2014) were annotated with genome release Creinhardtii_281_v5.5. To collect as many poly(A) sites as possible, we also identified additional poly(A) sites from RNA-seq data for rice (Davidson et al., 2012; Wang H. et al., 2015) and Medicago (Wang T. et al., 2015; Mertens et al., 2016).

A Perl script was written to extract the chromosome names, nucleotide contents, and chromosome lengths from a FASTA file of genome sequence and then insert the data into the database. For each species in the database, another Perl script parses the genome annotation in GFF format. Because a gene may contain more than one transcript, we proposed a method to determine unique gene models. If a region is not the same among different transcripts of the same gene, it is denoted as an AMB (AMBiguous) region (Figure 1A). The incorporation of an AMB region prevents double counting of poly(A) sites corresponding to multiple transcripts from the same gene. In addition, since current genome annotations of most species are largely based on protein-coding predictions and thus likely to lack 3′ UTR information, the genome annotation of each species was modified to better estimate the occurrence of poly(A) sites within 3′ UTRs. Therefore, genes that had no annotated 3′ UTR downstream from the ends of the protein-coding regions were extended by the average length of 3′ UTRs in the respective species. Also, genes that had annotated 3′ UTR were extended by an arbitrary length as described in previous studies (Shen et al., 2008a; Wu et al., 2011, 2014; Zhao et al., 2014). These steps can improve the “recovery” of poly(A) sites that fall within authentic 3′ UTRs. We used this modified version of genome annotation to better annotate poly(A) sites in a majority of PlantAPA modules, while the original genome annotation was also presented in the *PAC browse* module as an alternative way for users to display poly(A) sites.

### Extraction of poly(A) sites

A computational pipeline was implemented in Perl to detect poly(A) sites from next generation sequencing (NGS) (Wu et al., 2015a) or EST data (Figure 1B). Three kinds of sequences in FASTA or FASTQ format can be used, including reads with T stretches at the 5′ end (T-reads), reads with A stretches at the 3′ end (A-reads), and ESTs. For a FASTQ file, a quality control check using the fastq_quality_filter function in the FASTX Toolkit can be performed to remove low-quality reads. Next, reads with T stretch at the 5′ end or A stretch at the 3′ end are filtered and the A/T stretches trimmed off. Reads without any A/T stretch, or those shorter than a given length (e.g., 25 nt), are discarded. Then Bowtie (Langmead et al., 2009) or Bowtie 2 (Langmead and Salzberg, 2012) are used to map all qualified reads, and GMAP (Wu and Watanabe, 2005) is used to map all ESTs to the reference genome. After obtaining reads that can be uniquely mapped to the reference genome, coordinates of candidate poly(A) sites can be obtained. Finally, candidate poly(A) sites that represent possible internal priming by reverse transcriptase are discarded using a strategy similar to that described in our previous study (Wu et al., 2011). Briefly, genomic sequences around poly(A) sites (i.e., −10 to +10 nt) are inspected for continuous As > 5 nt, or As > 6 nt in any 10 nt window, and these sites are discarded from subsequent analyses. Further, to reduce the impact of microheterogeneity, a snowball-like method was adopted to cluster poly(A) tags within a given distance (default of 24 nt) into a poly(A) site cluster (PAC). The information of each PAC is then recorded, including the start and end coordinates, the number of poly(A) tags, and the coordinates of the dominant cleavage site. Finally, PACs are annotated based on the refined genome annotation to determine their genomic locations (3′ UTR, CDS, intron, AMB, or intergenic).

### Analysis of alternative polyadenylation

We designed several functions implemented in Perl and R languages to compare general usages of poly(A) sites and gene expression levels between samples, including identification of 3′ UTR lengthening and shortening, identification of non-canonical APA site switching genes, detection of differentially expressed (DE) genes and significant changes in PAC levels (Figure 1C). First, to make PACs from different samples comparable, read count of PACs can be normalized in various ways, including the TPM (Tag Per Million) method, or EdgeR (Robinson et al., 2010) and DEseq (Anders and Huber, 2010) methods provided in R packages.

To discover significant 3′ UTR shortening or lengthening, genes with at least two PACs in 3′ UTRs are considered. The global lengthening or shortening of each gene is calculated by detecting a trend association for two-way tables with ordered levels (Agresti, 1996; Fu et al., 2011). Given *s* libraries and a gene with *k* poly(A) sites, read count and transcript length of the *i*^*th*^ PAC in the *j*^*th*^ library are *N*_*ij*_(*i* = 1…*s, j* = 1.…*k*) and *L*_*j*_, respectively. Then, PACs in a gene are sorted by the 3′ UTR lengths, and the lengths are considered as scores. A table is constructed with each row denoting the indexes of samples *S*_*i*_ and each column denoting the scores. Each element in the table is the read count *N*_*ij*_. Then a chi-squared test for trend in proportions is performed using the R function prop.trend.test, and the Pearson correlation *r* is calculated using the number of reads in the table as the values and the scores for rows and columns as coordinates. The correlation *r* falls between −1 and 1. The larger the correlation is in absolute value, the farther the data fall from independence in the linear dimension. Finally, *p*-values are adjusted using Benjamin method implemented by the R function p.adjust, and genes with adjusted *p*-values smaller than a given threshold are those with significant 3′ UTR shortening or lengthening.

In addition to the PACs located in 3′ UTRs, recent studies have uncovered an increasing number of PACs located in non-3′ UTRs, e.g., introns or CDS. To study such unconventional PACs, we proposed a method to detect non-canonical APA site switching genes. First, if a gene has more than two PACs, then the top two supported by the greatest number of PATs were used (denoted as *PA*1 and *PA*2). Genes with both *PA*1 and *PA*2 located in 3′ UTRs are discarded. Then genes passing through the following filtering criteria are considered as potential APA switching instances (Mangone et al., 2010; Wu et al., 2011): (1) distance between *PA*1 and *PA*2 at least *D* nt; (2) total read count for a gene more than *N1*; (3) *PA*1:*PA*2 read count ratio more than *K* (*K* > 1)-fold in one sample and *PA*2:*PA*1 also more than *K* (*K* > 1)-fold in another sample; (4) difference in read counts between *PA*1 and *PA*2 more than *N2* within each library in which switching occurred; and (5) *p*-value of Fisher's exact test for *PA*1 and *PA*2 read counts between the two libraries less than a given significance level.

Studies have suggested that 3′ end sequencing is quantitative at the level of mRNA abundance (Lianoglou et al., 2013); thus, we sum up PATs in a gene as gene expression level to detect DE genes between samples. To identify significant changes in gene expression levels, we applied the EdgeR (Robinson et al., 2010) and DEseq (Anders and Huber, 2010) packages, both of which have been widely used for assessing differential gene expression from RNA-seq data. For each gene, both *p*-value and adjusted *p*-value are calculated, and the one with *p*-value or adjusted *p*-value smaller than a given threshold (e.g., 0.01) is identified as a DE gene. We also designed a pipeline integrating DEXseq (Anders et al., 2012) to identify significant changes in PAC usage between two samples. DEXseq was originally developed to detect differential exon usage between samples from RNA-seq data, but it has also been used to identify significant changes in 3′ UTR isoform expression in animals from 3′ seq data (Lianoglou et al., 2013). To detect PACs with differential usages, genes with at least two PACs are filtered, and the PACs in each gene are numbered. Then these PACs are used as inputs for DEXseq, and the output is a list of PACs with *p*-value smaller than a given significance level.

### Web server implementation

PlantAPA consists of web front-end applications, a back-end database, and a series of Perl scripts and R scripts used for extraction, visualization, and analysis of poly(A) sites. We constructed the interactive web interfaces based on jQuery (http://jquery.com) and other JavaScript libraries to improve user experience. In order to make web interfaces more interactive and dynamic, integrated AJAX (Asynchronous Javascript and XML) technology was used to communicate with background pages and upgrade the front interface without reloading the website. Several open source plug-ins were also incorporated in our server, such as E-Charts (http://echarts.baidu.com/) and jTable (http://www.jtable.org), to display data dynamically. The poly(A) site extraction pipeline is composed of several Perl scripts and alignment tools, such as Bowtie (Langmead et al., 2009) and GMAP (Wu and Watanabe, 2005). The APA analysis pipelines integrate several Perl scripts, R scripts, and R packages like DESeq (Anders and Huber, 2010), EdgeR (Robinson et al., 2010), and DEXSeq (Anders et al., 2012). The JBrowse genome browser (Skinner et al., 2009) is integrated to enable dynamic and fluid browsing of PACs associated with genomes, genes, transcripts, and annotations on the whole genome level. MySQL is used to implement the back-end database, which stores several tables in its schema to record the genome annotation, coordinates and annotation of PATs and PACs, dynamic usages of PACs across different conditions, gene functions, and GO terms for each species.

## Results

### General organization of PlantAPA

**Figure 2:**
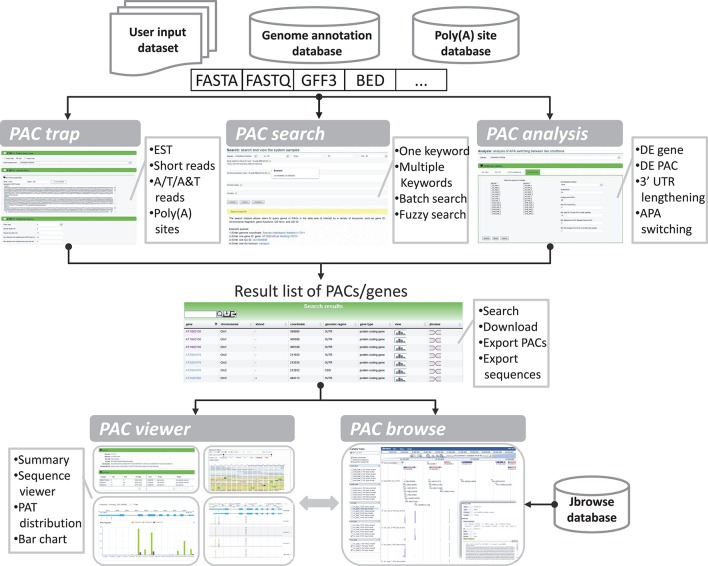
**General organization of PlantAPA**. PlantAPA mainly consists of five modules. Result PACs or genes from the *PAC trap* module, *PAC search* module, and *PAC analysis* module can be further visualized in *PAC viewer* or *PAC browse* modules. The comment box describes the main features or functions of the respective module.

The goal of the portal is to provide biologists with a convenient and meaningful platform for studying plant APA. To this end, we implemented five modules in PlantAPA (Figure 2), including extraction of PACs from user uploaded sequences (*PAC trap*), query of platform-collected PACs (*PAC search*), browsing PACs through JBrowse (*PAC browse*), analysis of APA between conditions (*PAC analysis*), and quantification and visualization of PACs (*PAC viewer*). In the *PAC trap* module, users can input their own short reads, ESTs, or known poly(A) sites to extract or upload their own PACs. Then users can visualize and analyze their uploaded PACs combined with stored PlantAPA PACs in various ways. In the *PAC search* module, users can query PACs by various kinds of keywords and choose to visualize detailed information, such as poly(A) signals, expression patterns, and sequences in the *PAC viewer* module. The *PAC browse* module integrates the Jbrowse genome browser to provide an interactive and graphical view of PACs on a genome-wide scale. In the *PAC analysis* module, users can compare general poly(A) site usage and changes in gene expression levels between libraries, combining user uploaded PACs, if any, and collected PlantAPA PACs. The *PAC viewer* module provides diverse representation modes to allow users to quantify and visualize PACs of a gene or intergenic region across different conditions.

### Datasets in PlantAPA

The datasets of samples from different cell and tissue types, or conditions, for four organisms, including Arabidopsis, Medicago, rice, and Chlamy, were processed and populated into the back-end database in PlantAPA. PlantAPA stores PACs involved in leaf, seed, and root tissues of wild type and *oxt6* mutant for Arabidopsis; leaf, hairy root, and root tissues for Medicago; flower, leaf, root, grain, and seed tissues for rice; and reads from Sanger, 454, Illumina, and poly(A) tag sequencing for Chlamy (Supplementary Table 1). In Arabidopsis, after pooling, and stringent filtering of reads from all samples, 69,577 PACs supported by ~99 million PATs were obtained (Table 1). More than half of these PACs are located in 3′ UTRs (32,719), supported by ~92% of PATs (Supplementary Figure 1A). CDS and introns harbor 15,229 PACs, and only 739 are located in 5′ UTRs. The pooled Medicago sample displays a similar distribution (Supplementary Figure 1B). The distribution of rice, however, slightly differs from Arabidopsis and Medicago (Supplementary Figure 1C). In rice, 3′ UTRs harbor 20% more PACs than the other two organisms. To account for this, current rice PACs were obtained from ESTs or RNA-seq data. Sanger sequencing is limited by throughput and scalability, thus inherently failing to discover unconventional PACs in CDS or introns, or PACs from lowly expressed genes. RNA-seq, however, is not powerful in detecting poly(A) sites because only a very small fraction of reads from RNA-seq can be used to quantify APA isoforms (Shi, 2012). The PACs from Chlamy were collected from multiple sequencing platforms (Zhao et al., 2014; Bell et al., 2016), exhibiting a distribution slightly different from that of the other three organisms (Supplementary Figure 1D). More than 25% of PACs are located in intergenic regions supported by 21% of PATs, and ~10% of PACs are located in introns, a much higher percentage than that of the other three organisms. The pooled PACs cover more than 13,000 protein coding genes in each of the four organisms, ranging from 13,263 to 22,542 (Supplementary Figure 2A). More than 60% of genes in all the four organisms exhibit at least two PACs (Supplementary Figure 2B).

**Table 1:** **Current PlantAPA datasets**.

**Species**	**Samples**	**PATs**	**PACs**	**Description**
*Arabidopsis thaliana*	20	98,781,156	69,577	Poly(A) sites from leaf, seed, and root tissues of WT and *oxt6* mutant by PAT-seq sequencing
*Oryza sativa*	10	1,236,410	55,465	Poly(A) sites extracted from ESTs and RNA-seq reads
*Medicago truncatula*	8	3,412,026	61,258	Poly(A) sites extracted from PAT-seq and RNA-seq reads
*Chlamydomonas reinhardtii*	4	13,536,005	45,372	Poly(A) sites extracted from reads from ESTs, 454, Illumina, and PAT-seq sequencing

We compared the PACs collected in PlantAPA with a previous dataset of Arabidopsis from direct sequencing (DRS) (Sherstnev et al., 2012). In total, 14,083 genes with poly(A) sites are present in both datasets (Supplementary Figure 3A), while 7645 genes are exclusively present in PlantAPA, and only 228 genes are unique in DRS. Apparently, PlantAPA contains substantially more expressed genes than DRS, which may be attributed to more diverse samples and more data collected in PlantAPA. To compare PACs between the two datasets, a marginal distance of 50 nt on both sides of a PAC was permitted to avoid the effect of cleavage site microheterogeneity. Up to 37,158 PACs were detected in both datasets, while a comparable number of PACs (15,695 and 12,758) were exclusively listed in PlantAPA or DRS (Supplementary Figure 3B). The exclusive PACs present in each dataset may be attributed to the biological variance of gene expression and the technical variance of different sequencing protocols. However, the large overlap among PACs detected by both datasets (>70%) underscores the reliability of PACs collected in PlantAPA.

### Extraction of PACs from uploaded sequences

The *PAC trap* module takes one, or more than one, file uploaded by a user as input, which allows file(s) of PAC coordinates or file(s) of short reads or ESTs in FASTA or FASTQ format. A label, such as library, sample name, tissue, or cell type, can be assigned to each uploaded file to help distinguish each input. If the input is a file with a PAC list, then users can skip the PAC extraction pipeline and switch to other modules for PAC visualization and analysis, using both the user- uploaded PACs and stored PlantAPA PACs. Otherwise, the user needs to choose the sequence type by clicking a drop-down box that shows different file types, including unknown, reads with T stretches at the 5′ end (T), and reads with A stretches at the 3′ end (A). If “unknown” is chosen, a sequence type (A or T) will be automatically assigned by comparing the frequency of occurrence of stretch A and stretch T in the uploaded file. Predefined sets of parameters are provided in the *PAC trap* module for general users. Nevertheless, users can modify the options to meet their specific needs, such as discarding low-quality sequences or not, minimum read length, alignment tools, removing internal priming candidates or not, and distance for clustering adjacent cleavage sites. Upon completion of the PAC extraction process (Figure 1B), users can download the PAC list directly from the web site onto their local computers. Also, additional information, such as mapping summary, single nucleotide composition around PACs, and top hexamers upstream of PACs, will be displayed in the result page, thus allowing readers the opportunity to evaluate their own data (Supplementary Figure 4). The output is provided in a tabulated list that allows users to sort, filter, and search individual PACs by several criteria, including, for example, locus ID, mapped chromosome start and end coordinates, and number of reads/ESTs. By following the web link on a particular PAC, users can continue to use other seamlessly integrated modules for PAC visualization and mining. Particularly, in the case of multiple input files, users can further compare poly(A) site usage between their input libraries or with libraries provided by PlantAPA through the *PAC analysis* module.

### Searching PlantAPA and exporting sequences

**Figure 3:**
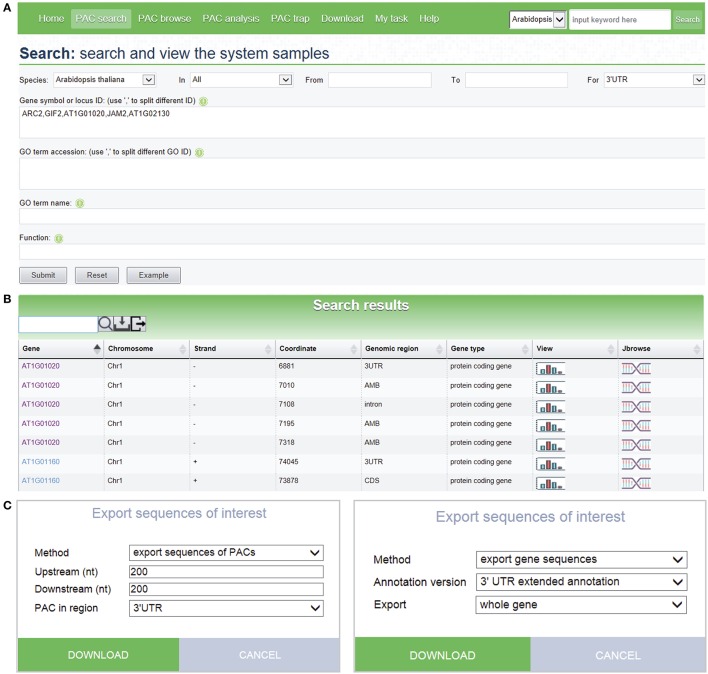
**Screen capture of the search page and a sample search result. (A)** Search interface designed to query PACs by combining a number of keywords. **(B)** Web page resulting from the batch search with search criteria shown in **(A)**. Each icon displayed in the “View” column of the result table links to a page that shows detailed information and PAC/PAT distributions of the corresponding gene or intergenic region. The icon in the “Jbrowse” column links to the PAC browser for browsing PACs and genes. **(C)** Pop-up windows designed to export sequences of PACs or genes with many options to control the output.

The *PAC search* module allows users to query genes or PACs in the datasets of interest. Searches can be restricted by various options to filter search results. Multi-keyword search by combining a number of keywords, such as chromosome fragment, genomic region, locus ID, gene name, and gene function, is permitted (Figure 3A). Batch search is also supported, where lists of locus IDs, gene names, and GO IDs can be provided as query input. An additional “fuzzy” search allows users to search database entries by a single keyword. The query output is a media page that lists all matched PACs in a dynamic table, where users can choose to view detailed information, such as poly(A) signals, sequences, and expression patterns, as well as graphics of PACs and the corresponding gene, by clicking the link on a PAC of interest. The entry of each gene provides links to other major databases like TAIR or Entrez Gene for more comprehensive information available for the gene of interest. A sample search result is shown in Figure 3B, in this case for the query shown in Figure 3A. It is noteworthy that the PAC list, rather than the gene list, is returned after searching, which enables users to retrieve the PACs in intergenic regions instead of genic regions. Recent studies have revealed the presence of poly(A) sites in intergenic regions which may be attributed to 3′ UTR extensions or novel transcript units (Wu et al., 2015b). Therefore, tabulating all PACs in genic, as well as intergenic, regions would facilitate the inspection of polyadenylation events associated with novel transcripts, lincRNAs, or antisense transcription. To further examine the search result, users can use the toolbar above the output table to locate specific entries by a keyword. Users can also export sequences of interest onto their local computers for other analytic purposes (Figure 3C). Sequences involving PACs or genes are exportable, including upstream and downstream sequences around PACs, sequences of PACs in a specific region (3′ UTR, 5′ UTR, CDS, intron, intergenic), sequences of genomic regions where PACs are located, and sequences of genes with PACs based on the original version or the extended version of genome annotation.

### Browsing PACs in the PAC browser

**Figure 4:**
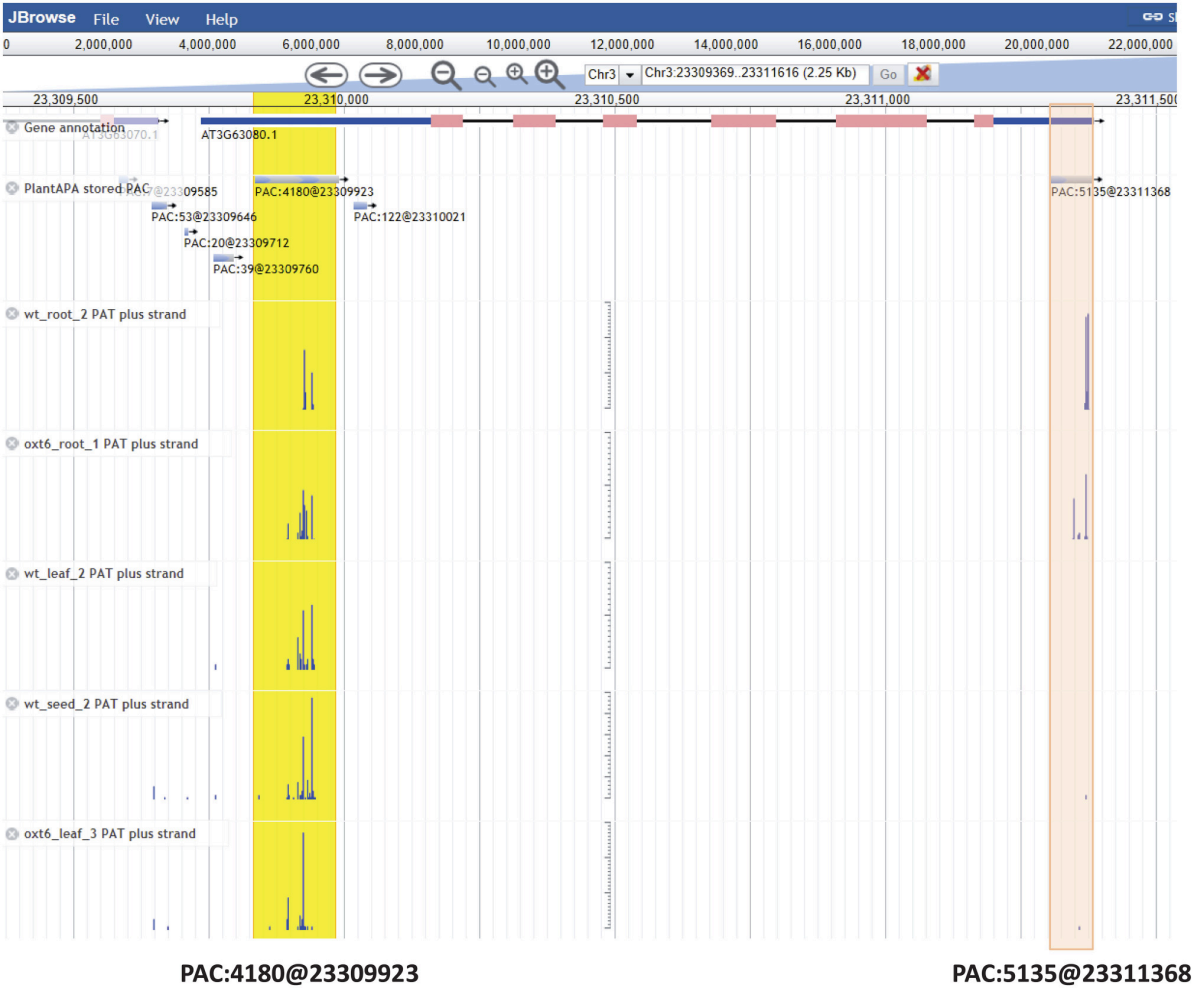
**Screen capture of display for PACs of Arabidopsis ***ATGPX5*** by the genome browser**. The PAC (PAC:5135@23311368) in 3′ UTR is specific to root tissue, which is highlighted in red. The PAC (PAC:4180@23309923) in 5′ UTR is highlighted in yellow, which is generally used by all tissues or conditions. For the mark denoting each PAC, e.g., PAC:5135@23311368, the number after the colon denotes the number of PATs, and the number after the @ symbol denotes the coordinate of this PAC.

The *PAC browse* module integrates a genome browser (Jbrowse) for dynamic browsing of PACs, together with genomes, genes, transcripts, and annotations, on a genome-wide scale. Users can have quick access to the PAC browser by clicking the “PAC browse” tab in the main menu or the “Jbrowse” link in a PAC list. One or more datasets from each plant species can be quickly loaded and graphically browsed online by selecting the checkboxes of datasets in the “Available Tracks” panel. Users can conduct a search for a gene or chromosome fragment to zoom in on particular genomic regions. Data tracks of PACs from different cells, tissues, or conditions can be displayed in sync with tracks of PATs (Supplementary Figure 5A), offering a more intuitive way to explore and compare the usage of PACs among different samples. As tracked and indicated in detailed layers (Figure 4), the PAC (PAC:5135@23311368) of Arabidopsis *ATGPX5* (Locus ID: *AT3G63080*) is a cluster which spans 76 bp from Chr3:23311308 to Chr3:23311383 with the reference coordinate at Chr3:23311368. This PAC seems to be specific to root tissue in that it has 2389 PATs in WT root and 2743 PATs in *oxt6* root, while only a few PATs (<3) were found in leaf or seed. Another PAC (PAC:4180@23309923) is located in 5′ UTR of the same gene, which spans 154 bp from Chr3:23309836 to Chr3:23309990 with the reference coordinate at Chr3:23309923 (Figure 4). This PAC has a total of 4180 PATs, which is generally used by all tissues or conditions. Users can download the data of one or more tracks onto their local computers, or choose to view detailed information of a gene or PAC (Supplementary Figure 5B) by clicking a PAC or gene model in the browser.

### Quantification and visualization of PACs across different conditions

**Figure 5:**
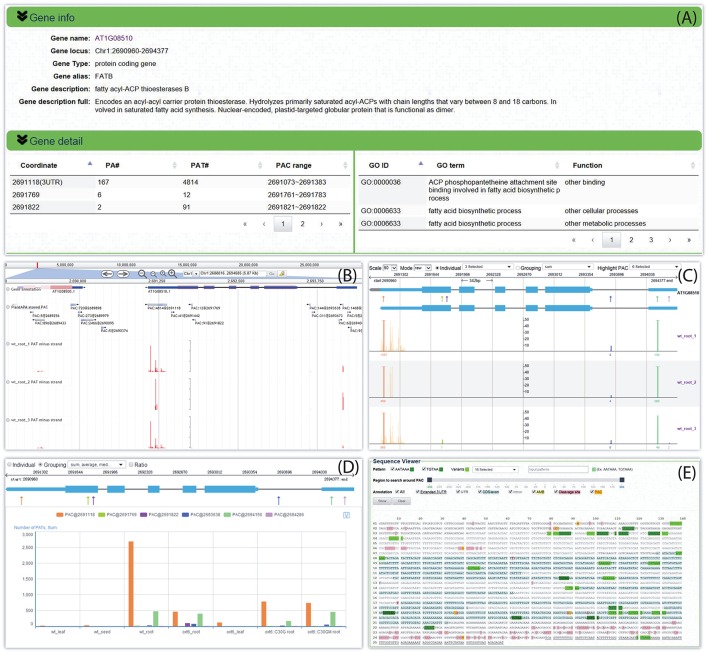
**Screen capture of the web page that reveals the dynamic usage of APA sites and expression pattern of ***FATB*** in Arabidopsis. (A)** Summary describes basic information about this gene and tabulates all PACs and associated GO terms. **(B)** Screenshot of a particular section of the PAC browser to show the intron-exon structure of *FATB* and the PAC/PAT tracks. **(C)** Graph presents both the gene model and the distributions of PACs/PATs across different samples. The top gene model is the refined one with extended 3′ UTR, and the bottom one is original. Each PAC is depicted as an arrow. Cleavage sites of a PAC are depicted in vertical lines with the same color as the PAC, and the height of the line represents the number of supporting PATs. The dominant cleavage site supported by maximum number of PATs in a PAC is marked by a thick line. If the number of PATs of the dominant cleavage site exceeds the maximum scale value of the vertical axis, a small horizontal line will be shown on the top of the thick line. A text label can be found under each dominant cleavage site to clearly indicate the total number of supporting PATs of the respective PAC. **(D)** Bar chart profiles the usage quantification of all PACs of *FATB* across different groups of samples. **(E)** Gene sequence of *FATB* annotated with gene model, poly(A) signals, and cleavage sites. This example can be shown at http://bmi.xmu.edu.cn/plantapa/sequence_detail.php?species=arab&method=search&seq=AT1G08510.

By following the web link on a particular PAC or gene, a web page of the *PAC viewer* module is returned for the user to inspect various graphics and detailed information of all PACs in a gene or intergenic region. Here we give an example for Arabidopsis *fatty acyl-ACP thioesterases B* (*FATB*, locus ID: *AT1G08510*). Summary information is given on the top of the web page, which describes basic information about this gene, such as gene alias or potential function, and then tabulates all six PACS and the associated GO terms (Figure 5A). Three kinds of graphs are displayed in the middle of the page to quantify and visualize the PAC/PAT distributions across samples. One is a screenshot of a particular section of the PAC browser to show the intron-exon structure of a gene, assuming that the PAC is located in the genic region, and the PAC/PAT tracks, thus allowing users to preview the gene model and locations of PACs before switching to the PAC browser (Figure 5B). Another dynamic graph presents both the gene model and the distributions of PACs/PATs across different samples in a more compact and intuitive way, where users can inspect the location, expression pattern, and usage of PACs in a gene, especially the selection of heterogeneous cleavage sites (Figure 5C). It is apparent that *FATB* possesses six PACs in different genic regions with distinct colors, two in 5′ UTR (PAC:9@2694286 and PAC:1468@2694156), one in intron (PAC:144@2693638), two in CDS (PAC:91@2691822 and PAC:12@2691769), and one in 3′ UTR (PAC:4814@2691118). Cleavage sites of a PAC are depicted in vertical lines with height representing the number of supporting PATs. The dominant cleavage site supported by the maximum number of PATs in a PAC is marked by a thick line. If the number of PATs of the dominant cleavage site exceeds the maximum scale value (default of 50) of the vertical axis, a small horizontal line will be shown on the top of the thick line. A text label is found under each dominant cleavage site to clearly indicate the expression level, i.e., total number of supporting PATs, of the respective PAC. Users can also view selected samples by choosing specific samples in the “Individual” drop-down list or group replicates within each individual sample by clicking the “Grouping” drop-down list. By clicking the “PAC usage” tab, an additional bar chart (Figure 5D) is presented to profile the usage quantification of all PACs of the queried gene across different groups of samples, providing a simple and direct way to compare the usage of PACs and determine ubiquitous or sample-specific PACs. By default, the bar chart displays the number of PATs of all samples. By checking the “Ratio” box, users can compare the usage of PACs within each sample to avoid great disparity in PAT number among samples. In addition to these diverse graphs, the bottom of the page presents the gene sequence annotated with gene model, poly(A) signals, and cleavage sites (Figure 5E). By default the most dominant poly(A) signal, AATAAA, and its 1 nt variants are scanned to obtain poly(A) signals. Users can also specify additional patterns to locate possible poly(A) signals. Users can set a region around poly(A) sites to narrow the scope of poly(A) signal search by dragging the slider. Further, users can choose to highlight genic regions, e.g., intron, exon, or 3′ UTR, cleavage sites, and poly(A) signals in different styles or colors in the corresponding sequence, facilitating manual inspection of the sequence of poly(A) sites in different genic locations. Particularly, heterogeneous cleavage sites of each PAC are in pink background, and the most dominant cleavage site in each PAC is denoted in red and highlighted in yellow.

PlantAPA uses unique gene models with AMB region and extended 3′ UTRs to display genomic context (Figure 1A). Here we take *HAP5C* as an example (Locus ID: *AT1G08970*) with PACs in AMB regions. This gene has four annotated transcripts (Supplementary Figure 6A), and four AMB regions were defined in the unique gene model (Supplementary Figure 6B). Three PACs are located in AMB regions (PAC:754@2884011, PAC:8290@2884248, and PAC:9@2884358), and one PAC is located at the boundary of stop codon and 3′ UTR (PAC:38@2883832). All these PACs are exclusively expressed in WT root and *oxt6* root. The one colored in purple (PAC: 8290@2884248) is the most dominant PAC, while the one in blue (PAC:9@2884358) is supported by only nine PATs and specific in WT root. The bar chart summarizes all PATs of individual groups to show the usage of PACs more clearly (Supplementary Figure 6C). PlantAPA details ambiguous regions and the related PACs instead of assigning a PAC to an arbitrary transcript of a gene with multiple annotated transcripts, which avoids incorrect assignment of PAC location. Nevertheless, manual checking is highly recommended to further investigate PACs in AMB regions. In addition to viewing PACs located in genic regions, intergenic PACs can also be searched and visualized in PlantAPA. Because an intergenic region may span a long range, the upstream 250 and downstream 250 bp region around the intergenic PAC was considered as a hypothetical gene for this PAC. Then users can choose to view this intergenic region in the same way as a gene. Here we give an example of an intergenic PAC, PAC:2009@17954540. This PAC is supported by 2009 PATs and is located in an intergenic region of chromosome 5 spanning from 17953254 to 17961285, which is ~1200 bp downstream of gene *AT5G44565* (Supplementary Figure 7A). Up to 11 PACs are located in this whole intergenic region, and two PACs are located in the displayed 500 bp intergenic region. PAC:2009@17954540 and another PAC 50 bp apart (PAC:5638@17954587) contain 100 heterogeneous cleavage sites with 7647 PATs (Supplementary Figure 7B). Interestingly, they are highly expressed in WT root and *oxt6* root, while rarely used in leaf or seed. By clicking the “PAC usage” tab, the usage of PACs across different groups of samples is clearly shown in a bar chart (Supplementary Figure 7C).

### Analysis of APA switching in different conditions

The *PAC analysis* module provides rich functionality for profiling the dynamic usage of PACs and exploring APA site regulation across different conditions, e.g., different samples, tissues, or developmental stages. Particularly, PlantAPA allows users to upload their own poly(A) sites or raw sequences, which enables users to analyze APA site switching with samples provided by the user or PlantAPA. By following the “PAC analysis” tab in the main menu, users can choose to generate lists of differentially expressed genes, PACs with differential usage, genes with 3′ UTR lengthening or shortening, and APA site switching genes. Two groups of samples, each with one or more than one condition, need to be selected first. Then, the raw count or normalized count calculated by different methods, such as TPM, EdgeR, and DESeq, can be specified. Additional parameters can be set for each assay, such as minimum number of PATs for prefiltering of PACs, significance level, and *p*-value adjusted method. To make the result statistically significant, a *p*-value or adjusted *p*-value will be calculated and assigned to each output PAC or gene. Users can download the output list to their local computer, or continue to inspect the resulting PAC or gene by clicking the link on a particular item on the list using the *PAC viewer* or the *PAC browse* module.

**Figure 6:**
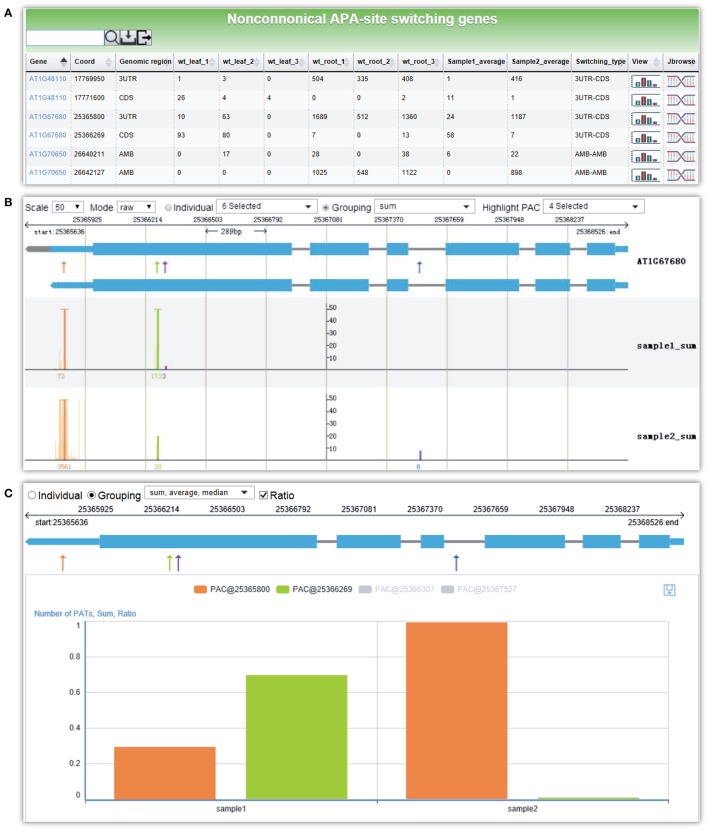
**Screen capture of the result page from ***PAC analysis*** and the display of the APA site switching gene ***AT1G67680*** in Arabidopsis. (A)** Result page of *PAC analysis* module for detecting non-canonical APA site switching genes. Three replicates of WT leaf sample and WT root sample in Arabidopsis were chosen, and default parameters were used. A list of 21 APA site switching genes was returned. **(B)** Screenshot of the graph that presents both the gene model and the distributions of PACs/PATs of one APA site switching gene, *AT1G67680*. “sample1_sum” and “sample2_sum” denote the sum of PATs of each PAC in the selected groups, i.e., WT leaf and WT root, respectively. **(C)** Bar chart profiles the usage quantification of the two PACs of *AT1G67680* involved in APA site switching. Shown are the ratios of PATs of the two PACs involved in APA site switching.

Here, we give an example for detecting non-canonical APA site switching genes between leaf and root in Arabidopsis. By selecting three replicates of WT leaf samples and WT root samples and applying default parameters, a result page is returned after a short period of running time. The output is a list of 21 APA site switching genes that possess at least two PACs with at least one PAC in a non-3′ UTR region and show significant difference in the uses of the two most abundant PACs between leaf and root (Figure 6A). Users can download the result list or export sequences of APA site switching genes or PACs onto their local computers. A summary table is displayed on the top of the result page, describing the parameters used and a summary of the result. By following the link on the gene list, users can switch to the *PAC viewer* module to view detailed information about a gene or PAC. For the example query of *AT1G67680*, the PAT figure (Figure 6B) shows usage quantification of four PACs in Arabidopsis (leaf and root), including one PAC in the annotated 3′ UTR (PAC@25365800, orange), two PACs located in CDS (PAC@25366269, green, and PAC@25366307, purple), and one PAC in intron (PAC@25367527, blue). According to the result table (Figure 6A), the PAC in CDS (PAC@25366269) and the one in 3′ UTR (PAC@25365800) are involved in the APA site switching. Obviously, PAC@25365800 in 3′ UTR is predominantly used in root, whereas PAC@25366269 in CDS is dominant in leaf. By choosing different statistical modes from the drop-down list, users can merge replicates or conditions of each group to simplify the display. In addition, the bar chart (Figure 6C) shows the expression pattern of PACs between the two samples. By checking the “Ratio” checkbox, the switching usage of the two PACs (PAC@25366269 and PAC@25365800) between leaf and root is apparent.

## Discussion

Increasing amounts of sequenced genomes and poly(A) sites in various plant species demand new methods and platforms for data access and mining. PlantAPA is intended to leverage our enormous wealth of existing poly(A) site data and genome annotations in plants by providing users with easy-to-use web services for the study of APA-related mechanisms and functions in plants. Like other APA-related web services (Müller et al., 2014; You et al., 2014), PlantAPA permits biologists to query and download poly(A) sites. With the user-friendly web interfaces and genome browser provided in PlantAPA, users can easily navigate, search, filter, and visualize all poly(A) sites. Considering data retrieval and visualization, PlantAPA has the following advantages over most other databases. First, to the best of our knowledge, PlantAPA is the only web service to date that focuses on poly(A) sites in plants. Second, diverse ways of graphical representation are presented, and a genome browser is integrated in PlantAPA for biologists to profile sequence features, heterogeneous cleavage sites, as well as quantify expression levels of poly(A) sites across different conditions. Interactive and dynamic graphics provide users with a convenient way to view selected samples or PACs of interest or to group replicates within each individual sample by taking average, median, or sum. Third, genome annotations were refined to determine AMB regions and extended 3′ UTR regions to enable more accurate mapping of PACs, and both original and modified versions of gene models are provided in PlantAPA. Therefore, users can query and visualize poly(A) sites in extended 3′ UTR regions and ambiguous regions owing to alternative transcription or RNA processing, as well as intergenic regions, thus enabling users to explore more deeply the mechanisms of polyadenylation events in previously overlooked genomic regions. Fourth, in addition to PACs clustered from the pooled data of each species, PlantAPA also preserves PACs and PATs of all replicates or data sources from a sample, enabling users to inspect the variance and reproducibility of PACs across different data sources or experiments. This is especially useful in determining candidate cleavage sites, PACs or genes with high reproducibility and reliability for confirmation by biological experiments.

APA has been shown to play an important role in the global control of gene expression in animals and plants. Tandem 3′ UTR switching can alter posttranscriptional regulation of gene expression in that it may lead to a gain or loss of miRNA binding sites. The APA site switching that occurs in CDS or introns may result in changes in translated proteins and their functions. Recent studies have investigated APA site switching, especially 3′ UTR lengthening or shortening, in various physiological states (Ji et al., 2009; Fu et al., 2011; Wu et al., 2011; Lianoglou et al., 2013; Miura et al., 2013). However, to date, neither comprehensive tools nor web services have been created to analyze APA site switching at the whole genome level. The study of APA site switching in non-3′ UTR regions is even more rare. In addition to PAC retrieval and visualization, PlantAPA also provides a user-friendly *PAC analysis* module for the exploration of APA site switching and differential gene expression between two conditions. To our knowledge, no previous web services have provided biologists with such functionality. The advantages of the *PAC analysis* module are as follows. First, diverse functions are provided for biologists to automatically analyze unconventional poly(A) site selections, 3′ UTR lengthening or shortening, differential uses of poly(A) sites, and differential gene expression between selected conditions, making PlantAPA convenient and powerful for the study of APA-directed gene regulations. Second, PlantAPA provides users with the capability to perform APA analysis with different parameter settings for prefiltering and custom analysis, which allows users to analyze PACs of interest and compare the results with different parameters for fine-tuning. Third, users may upload their own PACs from different samples and analyze them separately. Users can also analyze their PACs together with those collected in PlantAPA if they prefer to use a larger dataset or to compare their data with the existing data.

Although polyadenylation analysis remains a dominant approach for characterizing 3′ ends of various genomes, freely available and user-friendly bioinformatics tools for identifying poly(A) sites from raw sequences without the problems involved with software installation and configuration are rare. To the best of our knowledge, PlantAPA is the first public web service that provides the biology community with a unique module for the extraction of poly(A) sites from uploaded reads or ESTs. Different options can be tuned to customize the poly(A) site identification process. For example, to investigate the impact of clustering distance for grouping heterogeneous cleavage sites, a user might want to compare poly(A) site detection using two different criteria, e.g., 24 nts vs. 50 nts. Using two separate network sessions, a user can upload the same dataset independently to do comparative processing. Particularly, if the user already has one or more than one list of poly(A) sites, he/she can upload the list(s) to PlantAPA for data visualization and analysis. More importantly, PlantAPA allows users to make a comprehensive analysis of their own poly(A) sites and associated information combined with platform-stored poly(A) sites. With robust functionalities in poly(A) site query, visualization, and analysis, a user can easily appreciate the differences and relationships between their own poly(A) sites and the shared poly(A) sites in PlantAPA. Thus, PlantAPA provides users with an easy-to-use tool that can be customized and fully explored in terms of individual research demands.

We believe that PlantAPA will be a valuable addition to researchers engaged in the investigation of the polyadenylation and 3′ UTR, as well as those studying transcriptome and gene expression. Data updates will occur regularly upon the availability of new poly(A) site data. Continuous improvement will involve the retrieval of more sequencing data to obtain poly(A) sites, importation of data from additional organisms, and integration of new functionalities and tools to the web interface. Efforts are also underway to augment existing, related resources to provide the user with additional evidence for the usage and regulatory implications of poly(A) sites.

## Author contributions

QQL and XW conceived the study. XW developed the back-end pipelines contained in PlantAPA. YZ and XW implemented the web server. XW and QQL wrote the manuscript. All authors read and approved the final manuscript.

### Conflict of interest statement

The authors declare that the research was conducted in the absence of any commercial or financial relationships that could be construed as a potential conflict of interest.
