# Supplementary material for: PlantAPA: A Portal for Visualization and Analysis of Alternative Polyadenylation in Plants
Source: Front Plant Sci. 2016 Jun 21;7:889. doi: 10.3389/fpls.2016.00889 (PMC4914594; doi:10.3389/fpls.2016.00889)
Supplement: Supplementary file 1 [file Table1.DOCX]

Supplementary Table 1. Summary of data sets from all samples in PlantAPA website

| Lable | Tissue | Reference | PATs | PACs | Description |
| --- | --- | --- | --- | --- | --- |
| *Arabidopsis thaliana* (Genome annotation: TAIR 10; all raw data were sequenced by PAT-seq) | | | | | |
| wt leaf 1 | leaf | ([Thomas et al., 2012](#_ENREF_6)) | 1,055,461 | 11,570 | Plants were grown in soil in a climate-controlled growth room under short-day (8-h daylight) conditions, or under sterile conditions by germinating seeds, to capture as broad a range of poly(A) sites in leaves as possible. |
| wt leaf 2 | leaf | ([Wu et al., 2011](#_ENREF_10)) | 927,476 | 13,678 | ditto |
| wt leaf 3 | leaf | ([Thomas et al., 2012](#_ENREF_6)) | 24,269 | 3,989 | ditto |
| wt seed 1 | seed | ([Thomas et al., 2012](#_ENREF_6)) | 332,010 | 7,567 | RNA was isolated from dried Arabidopsis seed. |
| wt seed 2 | seed | ([Wu et al., 2011](#_ENREF_10)) | 2,230,409 | 15,106 | ditto |
| wt root 1 | root | ([Liu et al., 2014](#_ENREF_3)) | 16,232,735 | 53,850 | Total RNA were isolated from 10-day old roots. |
| wt root 2 | root | ([Liu et al., 2014](#_ENREF_3)) | 10,700,327 | 18,993 | ditto |
| wt root 3 | root | ([Liu et al., 2014](#_ENREF_3)) | 12,547,544 | 55,747 | ditto |
| *oxt6* root 1 | root | ([Liu et al., 2014](#_ENREF_3)) | 5,861,620 | 23,428 | A mutant deﬁcient in CPSF30 expression. Total RNA were isolated from 10-day old roots. |
| *oxt6* root 2 | root | ([Liu et al., 2014](#_ENREF_3)) | 10,174,020 | 18,923 | ditto |
| *oxt6* root 3 | root | ([Liu et al., 2014](#_ENREF_3)) | 5,653,903 | 48,191 | ditto |
| *oxt6* leaf 1 | leaf | ([Thomas et al., 2012](#_ENREF_6)) | 1,222,315 | 13,112 | A mutant deﬁcient in CPSF30 expression. Seedlings were germinated and grown in growth chambers set at 22 ℃ under continuous light. |
| *oxt6* leaf 2 | leaf | ([Thomas et al., 2012](#_ENREF_6)) | 74,991 | 6,950 | ditto |
| *oxt6* leaf 3 | leaf | ([Thomas et al., 2012](#_ENREF_6)) | 2,569,857 | 15,788 | ditto |
| *oxt6*::C30G 1 | root | ([Liu et al., 2014](#_ENREF_3)) | 2,277,879 | 24,681 | Transgenes that encode the wild-type AtCPSF30 were introduced into the oxt6 mutant that is deﬁcient in CPSF30 expression. Total RNA were isolated from 10-day old roots. |
| *oxt6*::C30G 2 | root | ([Liu et al., 2014](#_ENREF_3)) | 4,324,508 | 18,838 | ditto |
| *oxt6*::C30G 3 | root | ([Liu et al., 2014](#_ENREF_3)) | 5,510,456 | 47,620 | ditto |
| *oxt6*::C30GM 1 | root | ([Liu et al., 2014](#_ENREF_3)) | 5,360,339 | 23,679 | A mutant deficient in its interaction with calmodulin were introduced into the oxt6 mutant that is deﬁcient in CPSF30 expression. Total RNA were isolated from 10-day old roots. |
| *oxt6*::C30GM 2 | root | ([Liu et al., 2014](#_ENREF_3)) | 5,204,727 | 16,862 | ditto |
| *oxt6*::C30GM 3 | root | ([Liu et al., 2014](#_ENREF_3)) | 6,886,779 | 49,522 | ditto |
| *Oryza sativa* (Genome annotation: MSU v7; raw data were ESTs or RNA-seq reads) | | | | | |
| from EST | mix | ([Shen et al., 2008](#_ENREF_5)) | 57,852 | 28,616 | ESTs and partial or complete cDNA sequences, were collected from GenBank. |
| from  RNA-seq | mix | ([Davidson et al., 2012](#_ENREF_2)) | 47,180 | 11,870 | Poly(A) sites collected from RNA-seq reads of leaf, endosperm, embryo, seed, pistil, anther, and inflorescence tissues. |
| flower buds | WT | ([Wang et al., 2015a](#_ENREF_7)) | 153,823 | 25,115 | Poly(A) sites collected from RNA-seq reads. Seeds from the cultivated rice subspecies Oryza sativa L. ssp. Japonica cultivar Nipponbare were grown in a greenhouse in Singapore under natural light conditions. Flower buds were collected before ﬂowering. |
| flower | WT | ([Wang et al., 2015a](#_ENREF_7)) | 124,224 | 22,116 | Poly(A) sites collected from RNA-seq reads of flower tissue. |
| leaves  before flowering | WT | ([Wang et al., 2015a](#_ENREF_7)) | 139,209 | 21,345 | Poly(A) sites collected from RNA-seq reads of leaves. The before-ﬂowering sample was deﬁned as a mixture of different stages in a period from panicle initiation to 1 day before ﬂowering. |
| leaves  after flowering | WT | ([Wang et al., 2015a](#_ENREF_7)) | 127,962 | 21,147 | Poly(A) sites collected from RNA-seq reads of leaves. The after-ﬂowering sample was deﬁned as a mixture of different stages after the ﬂowering day. |
| roots  before flowering | WT | ([Wang et al., 2015a](#_ENREF_7)) | 168,028 | 23,692 | Poly(A) sites collected from RNA-seq reads of roots. The before-ﬂowering sample was deﬁned as a mixture of different stages in a period from panicle initiation to 1 day before ﬂowering. |
| roots  after flowering | WT | ([Wang et al., 2015a](#_ENREF_7)) | 114,200 | 19,770 | Poly(A) sites collected from RNA-seq reads of roots. The after-ﬂowering sample was deﬁned as a mixture of different stages after the ﬂowering day. |
| milk grains | WT | ([Wang et al., 2015a](#_ENREF_7)) | 163,445 | 15,720 | Poly(A) sites collected from RNA-seq reads of grains. |
| mature seeds | WT | ([Wang et al., 2015a](#_ENREF_7)) | 140,487 | 18,778 | Poly(A) sites collected from RNA-seq reads of seeds. |
| *Medicago truncatula* (Genome annotation: JCVI Medtr v4; raw data were sequenced from PAT-seq or RNA-seq) | | | | | |
| wt leaf | leaf | ([Wu et al., 2014](#_ENREF_9)) | 3,146,287 | 44,685 | RNA was isolated from the combined leaves and washed roots of 3-4 week-old nodule-free plants. |
| hairy root | root | ([Mertens et al., 2016](#_ENREF_4)) | 6,690 | 3,262 | Independent hairy root lines expressing a non-functional GUS gene. |
| leaf OS | leaf | ([Wang et al., 2015b](#_ENREF_8)) | 51,490 | 7,225 | mRNA isolated from leaves of M. truncatula seedlings treated with osmotic stress (OS). |
| leaf SS | leaf | ([Wang et al., 2015b](#_ENREF_8)) | 37,338 | 4,911 | mRNA isolated from leaves of seedlings treated with salt stress (SS). |
| leaf CK | leaf | ([Wang et al., 2015b](#_ENREF_8)) | 29,059 | 4,099 | mRNA isolated from leaves of seedlings treated with control (CK). |
| root OS | root | ([Wang et al., 2015b](#_ENREF_8)) | 42,947 | 9,413 | mRNA isolated from roots of seedlings treated with osmotic stress (OS). |
| root SS | root | ([Wang et al., 2015b](#_ENREF_8)) | 47,675 | 13,287 | mRNA isolated from roots of seedlings treated with salt stress (SS). |
| root CK | root | ([Wang et al., 2015b](#_ENREF_8)) | 50,540 | 9,877 | mRNA isolated from roots of seedlings treated with control (CK). |
| *Chlamydomonas reinhardtii* (Genome annotation: Creinhardtii 281 v55) | | | | | |
| From  illumina | mix | ([Zhao et al., 2014](#_ENREF_11)) | 622,248 | 35,630 | Illumina data were from DNAnexus (http://sra.dnanexus.com/) |
| from  454 | mix | ([Zhao et al., 2014](#_ENREF_11)) | 324,305 | 20,423 | 454 data were from DNAnexus (http://sra.dnanexus.com/) or Dr. Olivier Vallon from Institut de Biologie Physico-Chimmique. |
| from  EST | mix | ([Zhao et al., 2014](#_ENREF_11)) | 56,754 | 9,512 | ESTs were collected from both JGI and NCBI GenBank. |
| from  PAT-seq | mix | ([Bell et al., 2016](#_ENREF_1)) | 12,532,698 | 14,820 | Poly(A) sites in in cultures grown in four different media types: Tris-Phosphate (TP), Tris-Phosphate-Acetate (TAP), High-Salt (HS), and High-Salt-Acetate (HAS). |

# References

Bell, S.A., Shen, C., Brown, A., and Hunt, A.G. (2016). Experimental Genome-Wide Determination of RNA Polyadenylation in Chlamydomonas reinhardtii. *PloS one* 11(1)**,** e0146107. doi: 10.1371/journal.pone.0146107.

Davidson, R.M., Gowda, M., Moghe, G., Lin, H., Vaillancourt, B., Shiu, S.H., et al. (2012). Comparative transcriptomics of three Poaceae species reveals patterns of gene expression evolution. *Plant J.* 71(3)**,** 492-502. doi: 10.1111/j.1365-313X.2012.05005.x.

Liu, M., Xu, R., Merrill, C., Hong, L., Von Lanken, C., Hunt, A.G., et al. (2014). Integration of Developmental and Environmental Signals via a Polyadenylation Factor in Arabidopsis. *PloS one* 9(12)**,** e115779. doi: 10.1371/journal.pone.0115779.

Mertens, J., Pollier, J., Vanden Bossche, R., Lopez-Vidriero, I., Franco-Zorrilla, J.M., and Goossens, A. (2016). The bHLH Transcription Factors TSAR1 and TSAR2 Regulate Triterpene Saponin Biosynthesis in Medicago truncatula. *Plant Physiol.* 170(1)**,** 194-210. doi: 10.1104/pp.15.01645.

Shen, Y., Ji, G., Haas, B.J., Wu, X., Zheng, J., Reese, G.J., et al. (2008). Genome level analysis of rice mRNA 3'-end processing signals and alternative polyadenylation. *Nucleic Acids Res.* 36(9)**,** 3150-3161.

Thomas, P.E., Wu, X., Liu, M., Gaffney, B., Ji, G., Li, Q.Q., et al. (2012). Genome-Wide Control of Polyadenylation Site Choice by CPSF30 in Arabidopsis. *Plant Cell* 24(11)**,** 4376-4388. doi: 10.1105/tpc.112.096107.

Wang, H., Niu, Q.W., Wu, H.W., Liu, J., Ye, J., Yu, N., et al. (2015a). Analysis of non-coding transcriptome in rice and maize uncovers roles of conserved lncRNAs associated with agriculture traits. *Plant J* 84(2)**,** 404-416. doi: 10.1111/tpj.13018.

Wang, T., Liu, M., Zhao, M., Chen, R., and Zhang, W. (2015b). Identification and characterization of long non-coding RNAs involved in osmotic and salt stress in Medicago truncatula using genome-wide high-throughput sequencing. *BMC Plant Biol.* 15(131)**,** 015-0530. doi: 10.1186/s12870-015-0530-5.

Wu, X., Gaffney, B., Hunt, A., and Li, Q. (2014). Genome-wide determination of poly(A) sites in Medicago truncatula: evolutionary conservation of alternative poly(A) site choice. *BMC Genomics* 15(1)**,** 615. doi: 10.1186/1471-2164-15-615.

Wu, X., Liu, M., Downie, B., Liang, C., Ji, G., Li, Q.Q., et al. (2011). Genome-wide landscape of polyadenylation in Arabidopsis provides evidence for extensive alternative polyadenylation. *Proc. Natl. Acad. Sci. USA* 108(30)**,** 12533-12538. doi: 10.1073/pnas.1019732108.

Zhao, Z., Wu, X., Raj Kumar, P.K., Dong, M., Ji, G., Li, Q.Q., et al. (2014). Bioinformatics Analysis of Alternative Polyadenylation in Green Alga Chlamydomonas reinhardtii Using Transcriptome Sequences from Three Different Sequencing Platforms. *G3: Genes|Genomes|Genetics* 4(5)**,** 871-883. doi: 10.1534/g3.114.010249.
