# Supplementary material for: PlantAPA: A Portal for Visualization and Analysis of Alternative Polyadenylation in Plants
Source: Front Plant Sci. 2016 Jun 21;7:889. doi: 10.3389/fpls.2016.00889 (PMC4914594; doi:10.3389/fpls.2016.00889)
Supplement: Supplementary file 2 [file Image1.PDF]

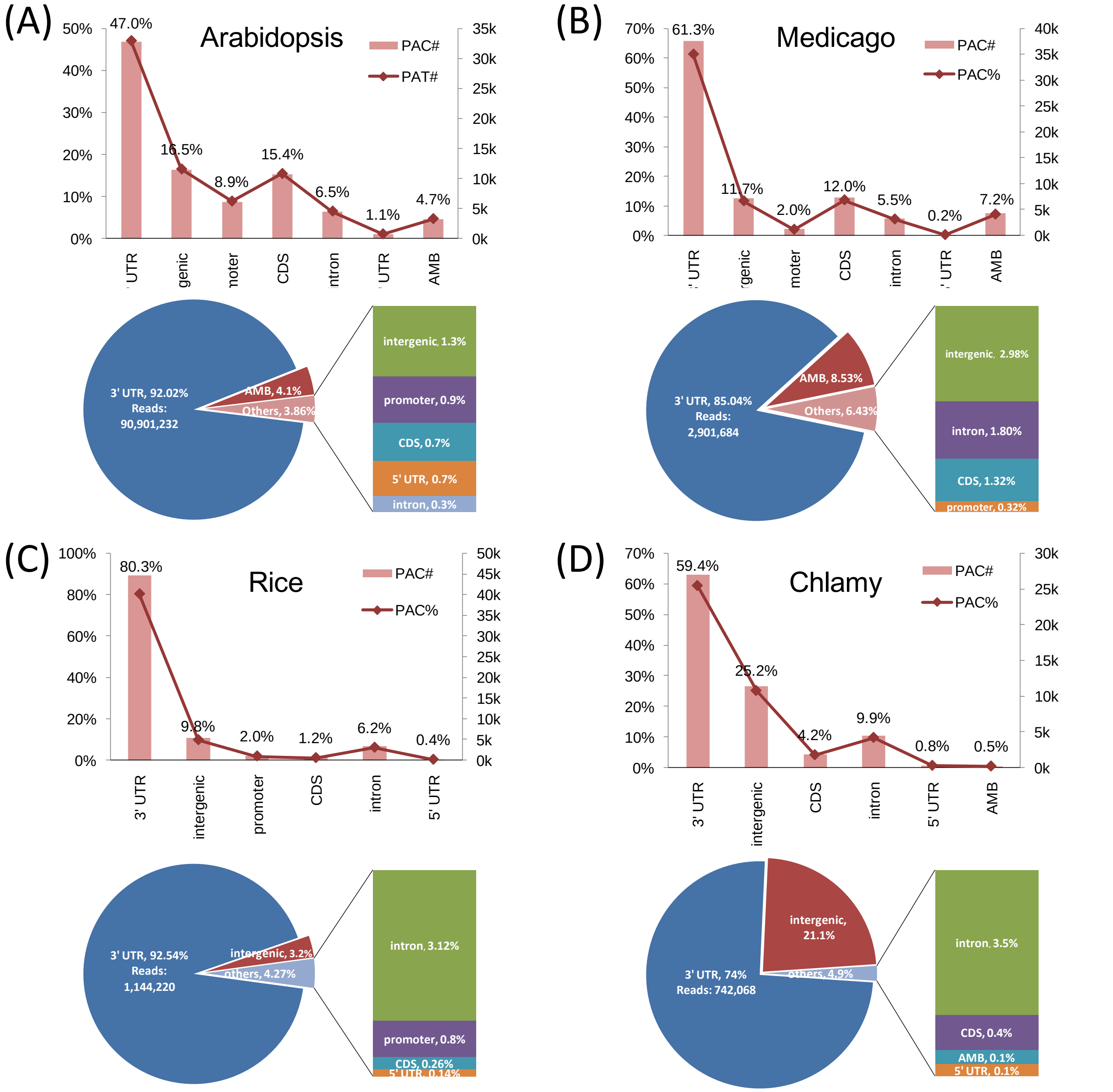

Supplementary Figure 1. Distributions of PACs (top, bar chart) and PATs (bottom, pie chart) in different genomic regions in the four organisms, Arabidopsis (A), Medicago (B), rice (C), and Chlamy (D). The bar chart presents the distribution of PACs in different genomic regions, including 3' UTR, intergenic, promoter, CDS, intron, 5' UTR, and AMB (Ambiguous region). The left axis represents the percentages of PACs in different regions; the right axis represents the number of PACs. The pie chart displays the distribution of PATs in different genomic regions. The number of PATs in 3' UTR is provided; only percentages of PATs are shown for other regions. PAC, poly(A) site cluster; PAT, poly(A) tag.
