# Supplementary material for: PlantAPA: A Portal for Visualization and Analysis of Alternative Polyadenylation in Plants
Source: Front Plant Sci. 2016 Jun 21;7:889. doi: 10.3389/fpls.2016.00889 (PMC4914594; doi:10.3389/fpls.2016.00889)
Supplement: Supplementary file 3 [file Image2.PDF]

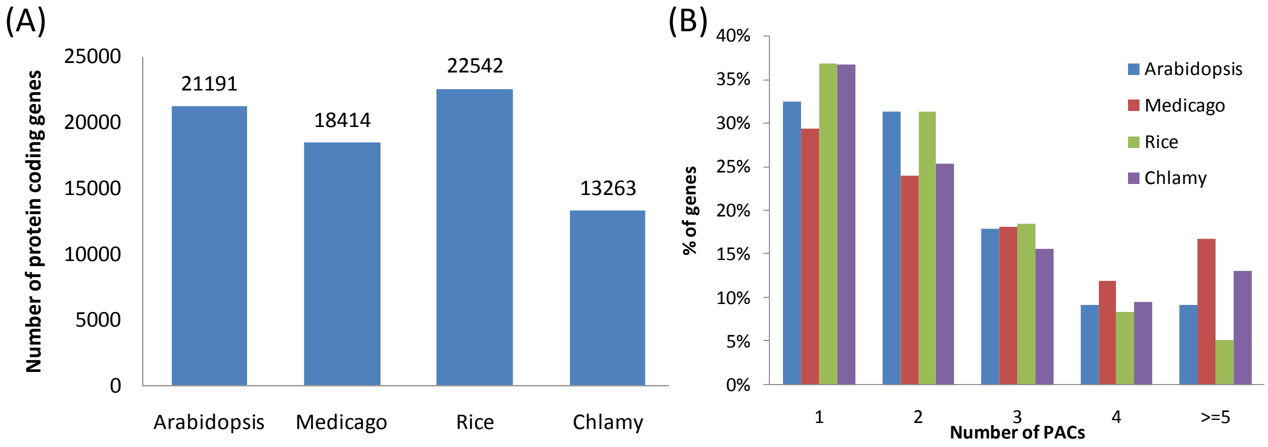

Supplementary Figure 2. Number of expressed genes and APA genes. (A) Number of protein coding genes with at least one poly(A) site in the four organisms. (B) Number of genes with different number of poly(A) sites in the four organisms. For example, the value of Y-axis corresponding to the number '3' in the X-axis means the percentage of genes with total three poly(A) sites.
