# Supplementary material for: PlantAPA: A Portal for Visualization and Analysis of Alternative Polyadenylation in Plants
Source: Front Plant Sci. 2016 Jun 21;7:889. doi: 10.3389/fpls.2016.00889 (PMC4914594; doi:10.3389/fpls.2016.00889)
Supplement: Supplementary file 4 [file Image3.PDF]

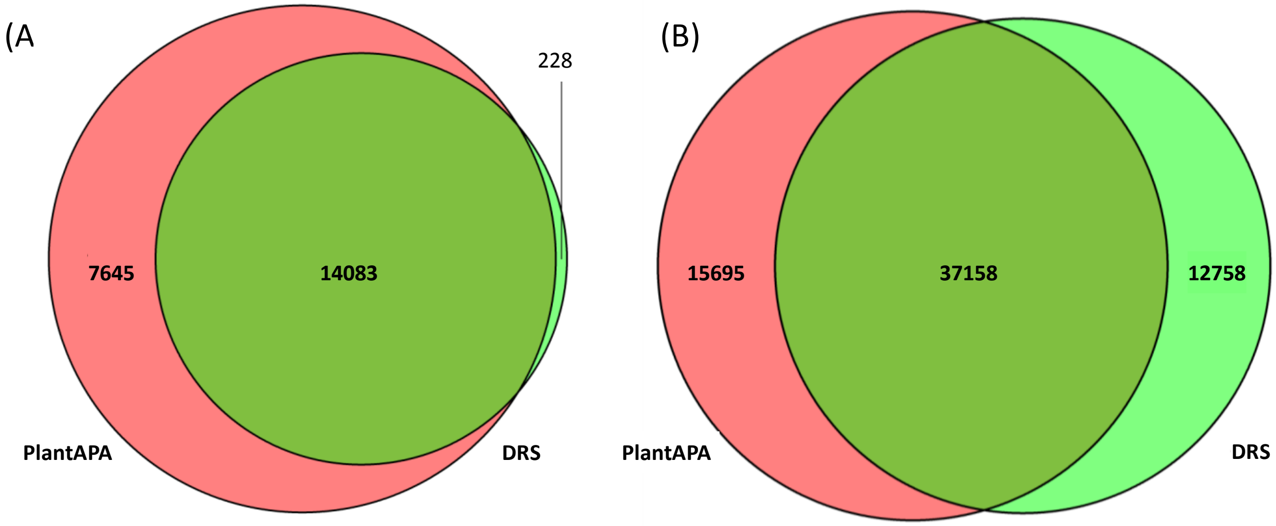

Supplementary Figure 3. Comparison of PACs collected in PlantAPA with a previous data set of Arabidopsis from direct sequencing (DRS). (A) Venn diagram of the number of genes with poly(A) site in two data sets. In total, 14,083 genes with poly(A) sites are present in both datasets, while 7645 genes are exclusively present in PlantAPA, and only 228 genes are unique in DRS. (B) Venn diagram of the number of poly(A) site clusters in two data sets. Up to 37,158 PACs were detected in both datasets, while a comparable number of PACs (15,695 and 12,758) were exclusively listed in PlantAPA or DRS. Here PACs located in intergenic regions are not included.
