# Supplementary material for: PlantAPA: A Portal for Visualization and Analysis of Alternative Polyadenylation in Plants
Source: Front Plant Sci. 2016 Jun 21;7:889. doi: 10.3389/fpls.2016.00889 (PMC4914594; doi:10.3389/fpls.2016.00889)
Supplement: Supplementary file 5 [file Image4.PDF]

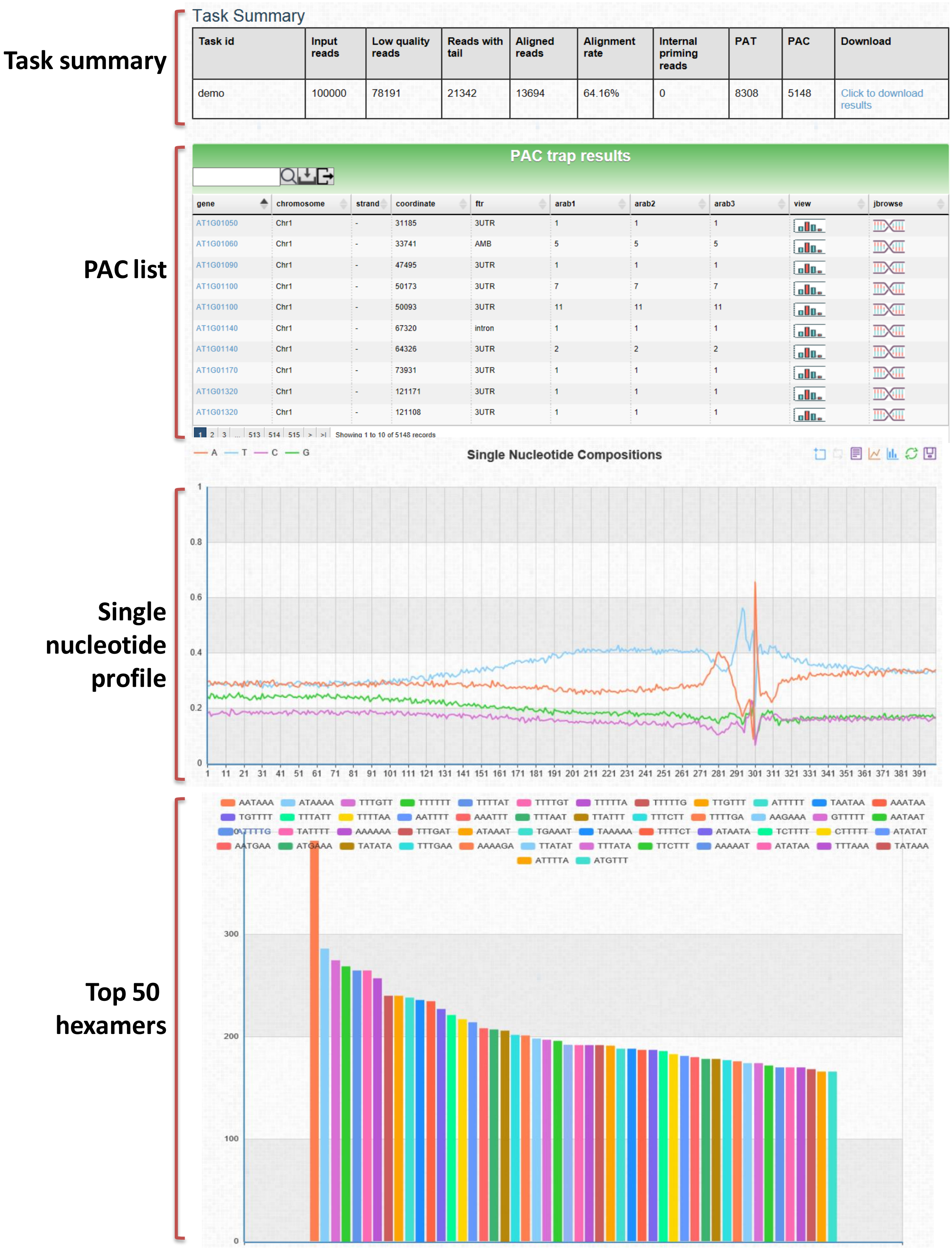

Supplementary Figure 4. Screen capture of the result of the *PAC trap* module. This example can be shown via the URL http://bmi.xmu.edu.cn/plantapa/show_result.php. The task summary panel

provides the mapping summary, such as number of input reads, number of final detected PATs and PACs. The PAC list presents the list of detected PACs; users can choose to view detailed information of a gene or PAC by clicking the respective icon. Single nucleotide compositions around PACs are displayed in the single nucleotide profile panel. Y-axis values are the fractional nucleotide content at each position (plotted along the x-axis); “0” on the X-axis denotes the actual poly(A) site. The bottom panel shows the number of top 50 hexamers in the near upstream region (-35 to -5) of PACs.
