# Supplementary material for: PlantAPA: A Portal for Visualization and Analysis of Alternative Polyadenylation in Plants
Source: Front Plant Sci. 2016 Jun 21;7:889. doi: 10.3389/fpls.2016.00889 (PMC4914594; doi:10.3389/fpls.2016.00889)
Supplement: Supplementary file 6 [file Image5.PDF]

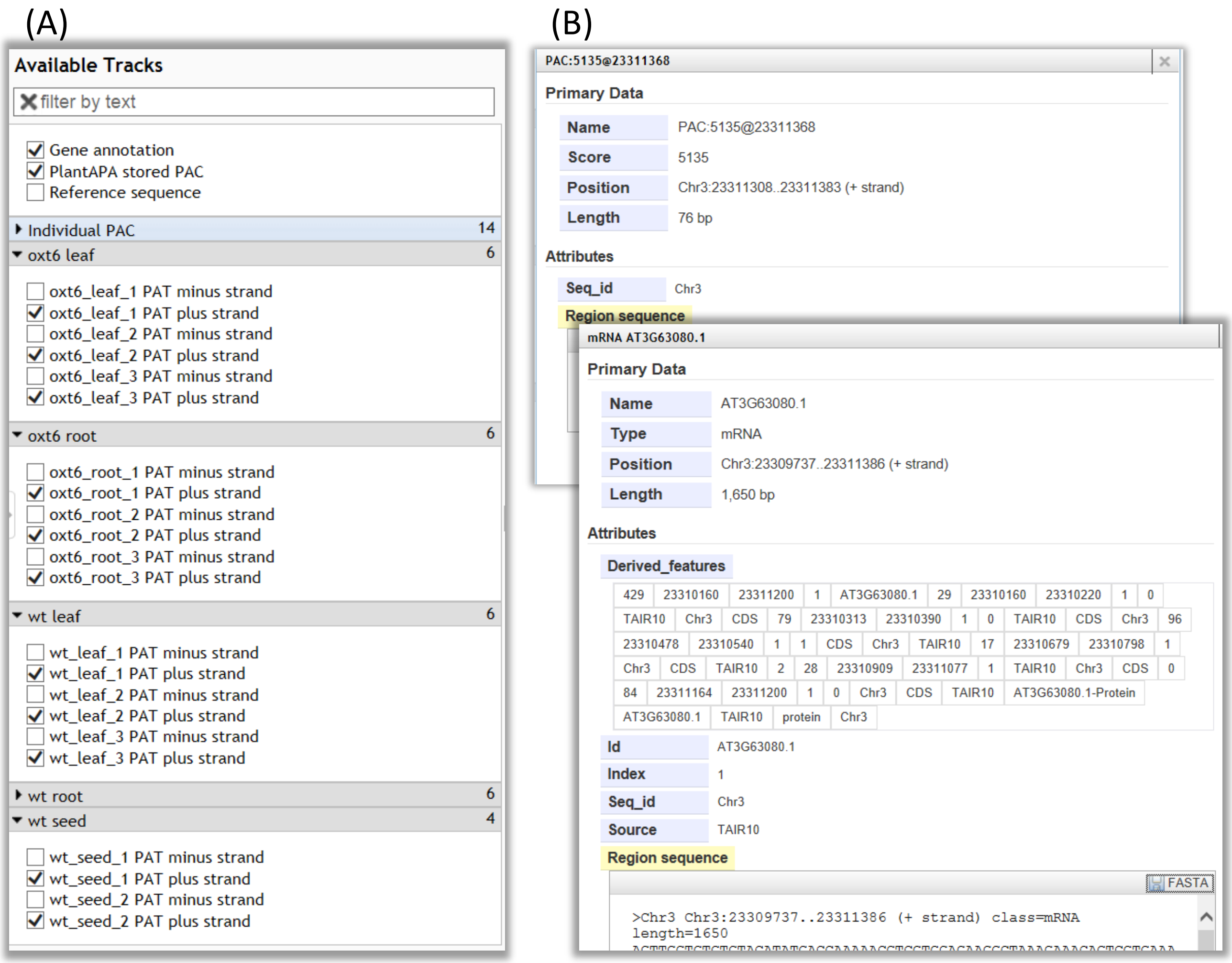

Supplementary Figure 5. PAC browser. (A) Data tracks of PACs from different cells, tissues or conditions can be displayed in sync with tracks of PATs. (B) Detailed information of a PAC or mRNA by clicking a PAC or mRNA in the browser. This example can be shown via the URL http://bmi.xmu.edu.cn/jbrowse/?data=data%2Farab&loc=Chr3%3A23309591..23312076&tracks=Gene%20annotation%2CPlantAPA%20stored%20PAC%2Cwt_root_1%20PAT%20plus%20strand%2Cwt_root_2%20PAT%20plus%20strand%2Cwt_leaf_1%20PAT%20plus%20strand%2Cwt_leaf_2%20PAT%20plus%20strand%2Cwt_seed_2%20PAT%20plus%20strand&highlight=Chr3%3A23309836..23309990.
