# Supplementary material for: PlantAPA: A Portal for Visualization and Analysis of Alternative Polyadenylation in Plants
Source: Front Plant Sci. 2016 Jun 21;7:889. doi: 10.3389/fpls.2016.00889 (PMC4914594; doi:10.3389/fpls.2016.00889)
Supplement: Supplementary file 7 [file Image6.PDF]

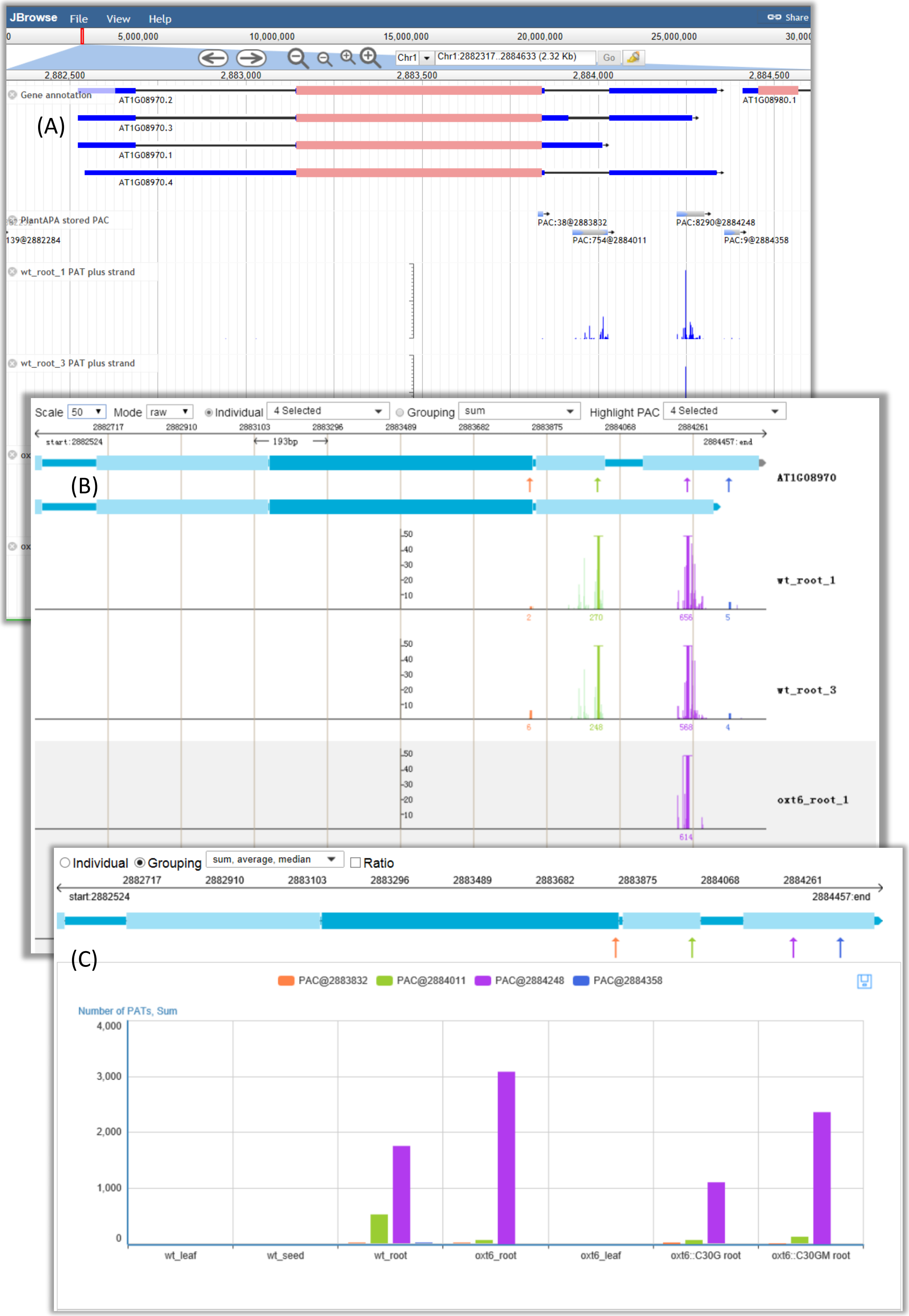
(A)
JBrowse File View Help
Share
Chr1:2882317..2884633 (2.32 Kb)
Gene annotation
AT1G08970.2
AT1G08970.3
AT1G08970.1
AT1G08970.4
AT1G08980.1
PlantAPA stored PAC
PAC:38@2883832
PAC:754@2884011
PAC:8290@2884248
PAC:9@2884358
wt_root_1 PAT plus strand
wt_root_3 PAT plus strand
(B)
Scale 50 Mode raw Individual 4 Selected Grouping sum Highlight PAC 4 Selected
start:2882524
193bp
2884457:end
AT1G08970
wt_root_1
wt_root_3
oxt6_root_1
(C)
Individual Grouping sum, average, median Ratio
start:2882524
2884457:end
PAC@2883832 PAC@2884011 PAC@2884248 PAC@2884358
Number of PATs, Sum
wt_leaf
wt_seed
wt_root
oxt6_root
oxt6_leaf
oxt6::C30G root
oxt6::C30GM root

Supplementary Figure 6. Visualization of PACs in AMB regions. (A) Gene model of *AT1G08970* with four transcripts. (B) AMB regions defined in the unique gene model and the distribution of PACs in *AT1G08970*. The top gene model is the refined gene model with extended 3' UTR and AMB regions and the bottom one is the original gene model. (C) The bar chart summarizes all PATs of individual samples to show the usage of PACs in *AT1G08970*. This example can be shown via the URL http://bmi.xmu.edu.cn/plantapa/sequence_detail.php?species=arab&method=search&seq=AT1G08970
