# Supplementary material for: PlantAPA: A Portal for Visualization and Analysis of Alternative Polyadenylation in Plants
Source: Front Plant Sci. 2016 Jun 21;7:889. doi: 10.3389/fpls.2016.00889 (PMC4914594; doi:10.3389/fpls.2016.00889)
Supplement: Supplementary file 8 [file Image7.PDF]

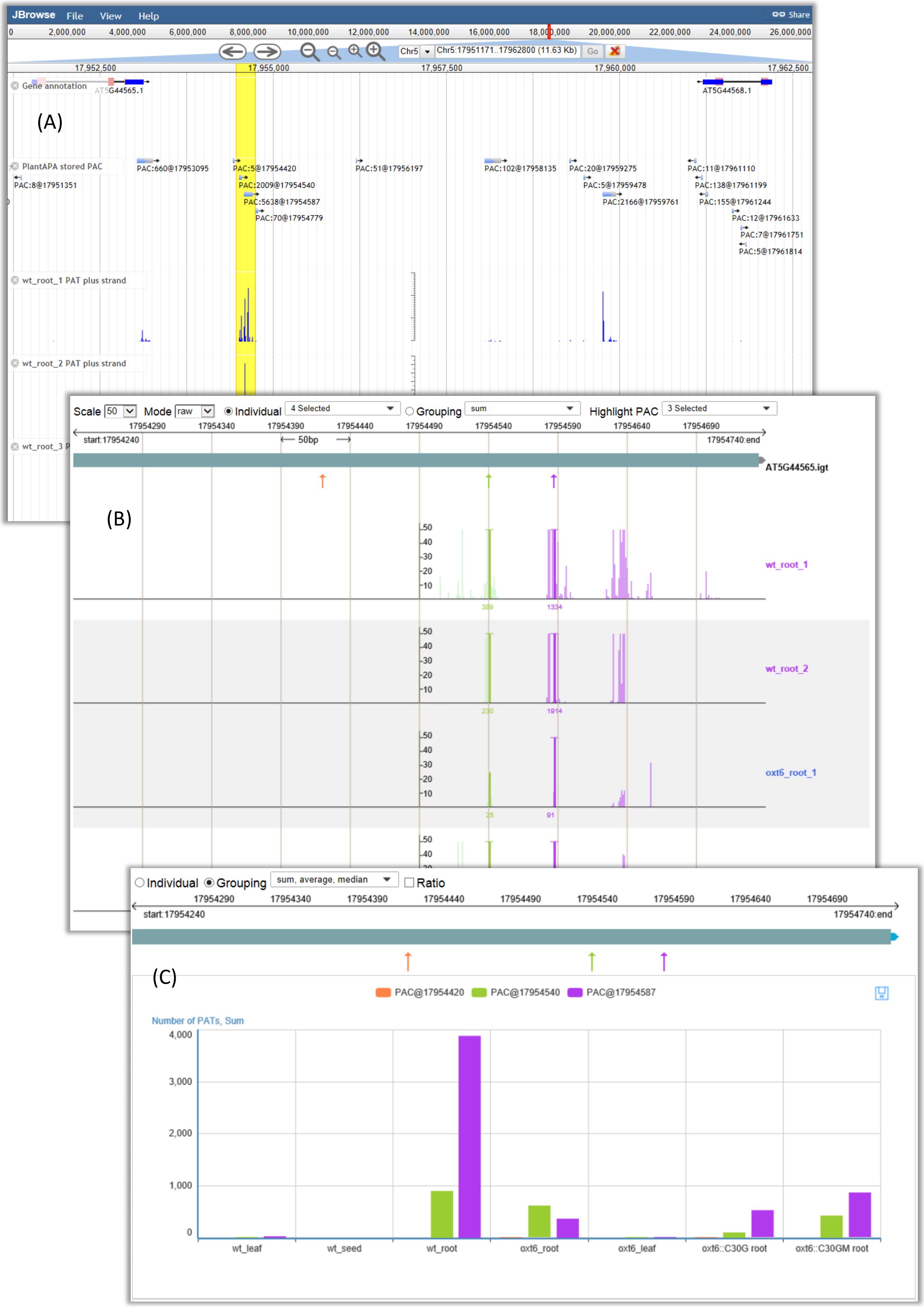

Supplementary Figure 7. Visualization of PACs in an intergenic region. (A) The 500 bp intergenic region of chromosome 5 spanning from 17954240 to 17954740 and the PACs in this region. PAC:

2009@17954540 and PAC:5638@17954587 are highlighted. (B) The upstream 250 and downstream 250 bp region around PAC:2009@17954540, and the PAT distributions of PACs in this region. (C) The bar chart that displays the usage of PACs across different conditions. This example can be shown via the URL http://bmi.xmu.edu.cn/plantapa/sequence_detail.php?species=arab&seq=AT5G44565.igt&strand=1&method=search&flag=intergenic&coord=17954540
